# Orthogonal Radiographs After Shoulder Reduction: Are We Meeting the Standard?

**DOI:** 10.7759/cureus.94814

**Published:** 2025-10-17

**Authors:** Uday Mahajan, Muhammad Yousaf, Kehinde Jinadu, Kashif Memon

**Affiliations:** 1 Trauma and Orthopaedics, Queen Elizabeth Hospital Birmingham, Birmingham, GBR; 2 Urology, Queen Elizabeth Hospital Birmingham, Birmingham, GBR

**Keywords:** emergency medicine and trauma, orthogonal views, plain radiography, quality improvement, shoulder anterior dislocation

## Abstract

Background

Anterior shoulder dislocation is a common emergency presentation. Post-reduction radiographs are essential to confirm concentric reduction and exclude associated injuries. Guidelines from the British Elbow and Shoulder Society (BESS) and local hospital policy recommend orthogonal views, including anteroposterior and either axillary or scapular Y projections. Failure to obtain adequate imaging risks missed malreduction, overlooked fractures, and unnecessary repeat imaging.

Methods

We conducted a retrospective service evaluation of 22 consecutive patients with anterior shoulder dislocation managed in the emergency department of a single tertiary centre. Radiographic adequacy was defined as the presence of orthogonal post-reduction views in accordance with BESS and local guidance. Data collected included demographics, initial and post-reduction imaging, adequacy of views, whether inadequacies were recognised or acted upon by resident doctors, repeat imaging, and additional investigations.

Results

Only five of 22 patients (22.7%, 95% CI 10.1-43.4) received adequate orthogonal post-reduction radiographs. Inadequate imaging occurred in 17 of 22 patients (77.3%), of which only two cases were recognised by resident doctors (11.8% of documented inadequate cases). No repeat imaging was requested in the emergency department to address inadequate films. Two patients underwent further radiographs later during inpatient admission for social reasons, unrelated to adequacy. Although discharge flow was not interrupted, this reflected a failure to recognise inadequacy at the time of ED care. Additional imaging with MRI, MRI arthrogram, or CT was obtained in several cases as part of subsequent outpatient management.

Conclusion

Compliance with post-reduction imaging standards for shoulder dislocation was poor, and inadequate films were rarely recognised by resident doctors. This represents both a guideline compliance gap and a training issue in radiograph interpretation. Ensuring orthogonal post-reduction views is a basic, low-cost intervention that can improve patient safety and diagnostic certainty. Targeted education and reinforcement of departmental policy, followed by re-audit, are recommended.

## Introduction

Anterior shoulder dislocation is one of the most common large-joint dislocations presenting to the emergency department [1]. Prompt recognition, safe reduction, and confirmation of joint congruity are essential to avoid complications such as recurrent instability, malreduction, and associated fractures [2]. Radiography is central to this process. Standard practice, supported by the British Elbow and Shoulder Society (BESS) guideline and local hospital policy, requires orthogonal views after reduction, typically an anteroposterior (AP) film supplemented by either an axillary or scapular Y view [3]. These projections confirm concentric reduction and allow detection of associated injuries such as Hill-Sachs lesions or glenoid rim fractures.

Despite these clear recommendations, clinical practice often falls short. Obtaining orthogonal views in the acute setting can be technically challenging, particularly in patients experiencing severe pain or limited range of motion. Emergency department pressures, variable radiographer familiarity, and lack of confidence among junior doctors in judging adequacy may further contribute to inconsistency [4-6]. Failure to obtain appropriate views carries important risks: malreduction may go unrecognised, associated injuries may be overlooked, and patients may require subsequent repeat imaging, delaying discharge and consuming additional resources [7].

The primary objective of this study was to evaluate the adequacy of post-reduction radiographs in anterior shoulder dislocation, and the secondary objectives were to assess whether inadequacies were recognised by resident doctors and to consider the potential implications for patient safety and service efficiency.

## Materials and methods

This was a retrospective service evaluation conducted in the Emergency Department of Queen Elizabeth Hospital, Birmingham, examining consecutive presentations of acute glenohumeral dislocation between March 2025 and June 2025. Eligible cases were adults (≥16 years) with a confirmed shoulder dislocation managed in the ED and undergoing reduction during the index visit. Exclusions were periprosthetic dislocations, fracture-dislocations requiring immediate operative management, chronic or irreducible dislocations, multidirectional instability, atraumatic dislocations and repeat attendances for the same dislocation episode.

Radiographic adequacy was defined according to BESS guidance and the local hospital policy, as the presence of orthogonal post-reduction views sufficient to confirm concentric reduction and exclude associated injury. Adequacy required an AP shoulder view plus either a true axillary view or a scapular Y view that clearly demonstrated the humeral head-glenoid relationship. AP-only were classified as inadequate. Pre-reduction imaging was recorded but not used for the primary adequacy outcome.

For each case, data were collected on patient demographics, side and mechanism of injury, whether the dislocation was first-time or recurrent, and the method of sedation or analgesia. The type of pre- and post-reduction radiographs obtained was recorded, along with any need for repeat imaging. The primary outcome was the proportion of cases with adequate orthogonal post-reduction radiographs. Secondary outcomes included whether inadequacy was recognised or acted upon by the resident doctor, as documented in clinical notes, imaging requests, or escalation. Additional outcomes were the consequences attributable to inadequate imaging, including further imaging requests, delays to discharge, the need for patients or staff to return for additional radiographs, and any missed malreductions or associated fractures identified only after repeat imaging.

Data were obtained from the electronic health record, ED notes, and the radiology picture archiving and communication system (PACS). A standardised data collection form was piloted on three cases and then applied to the full cohort of the most recent 22 eligible cases. Radiograph adequacy was assessed by the lead investigator and verified by a second reviewer familiar with shoulder radiographs, with discrepancies resolved by consensus. Missing data were left as missing and reported.

Statistical analysis was descriptive. Categorical variables are presented as counts and percentages. The primary outcome, the proportion of patients with adequate post-reduction radiographs, is reported with a 95% confidence interval calculated using the Wilson score method for binomial proportions. Continuous variables are summarised as mean with standard deviation or median with interquartile range, according to distribution. No hypothesis testing was pre-specified given the service evaluation design and small sample size. All analyses were performed using Microsoft Excel (Microsoft Corporation, Redmond, WA, USA).

This project was registered as a service evaluation and quality improvement activity with the Trust governance team and therefore did not require Research Ethics Committee review. Patient identifiers were not recorded on the study database. The intended next step is an educational intervention for emergency department clinicians and radiographers on the importance of orthogonal shoulder views, followed by a re-audit using the same methodology.

## Results

Twenty-two patients with anterior glenohumeral dislocation were included. Post-reduction radiography comprised a single view in 17 of 22 cases (77.3%) and two views in five of 22 (22.7%). Using the predefined adequacy standard (orthogonal post-reduction views), five of 22 cases (22.7%) had adequate imaging; the estimated proportion of adequate imaging was 22.7% (95% CI 10.1-43.4), whereas 17 of 22 (77.3%) were inadequate. In all cases, radiograph requests did not explicitly specify the requirement for two orthogonal post-reduction views.

Among the 17 inadequate cases, resident recognition of inadequacy was documented in two, yielding a recognition rate of 11.8% within the subset with documentation. In no case was repeat imaging obtained while the patient remained in the emergency department to correct inadequate views. Two patients underwent repeat radiography later during an inpatient stay for social admission reasons; these repeats were not undertaken to address radiographic inadequacy. Consequently, there were no documented flow interruptions or discharge delays attributable to repeat imaging requested in the ED (Table 1).

**Table 1:** Baseline characteristics and imaging outcomes (n = 22)

Variable	Value
Age (years), mean ± SD	36.6 ± 21.4
Sex	
– Male, n (%)	17 (77.3%)
– Female, n (%)	5 (22.7%)
Mechanism of injury	
– Fall, n (%)	8 (36.4%)
– Sports injury, n (%)	8 (36.4%)
– Road traffic collision (RTC), n (%)	4 (18.2%)
– Assault, n (%)	2 (9.1%)
First-time dislocation, n (%)	16 (72.7%)
Recurrent dislocation, n (%)	6 (27.3%)
Fracture-dislocation, n	2
Adequate post-reduction radiographs, n (%)	5 (22.7%)
Inadequate post-reduction radiographs, n (%)	17 (77.3%)

Initial diagnostic imaging before reduction most comprised two views (20/22, 90.9%). Fracture-dislocation was present in two of 22 (9.1%) and first-time dislocation in 17 of 22 (77.3%). Additional cross-sectional imaging was frequent in subsequent care pathways: MRI in seven cases, MRI arthrogram in two, CT in one, and combined CT+MRI in one; MRI was planned but not completed in four cases. One patient was recorded with a complication (“mild anterior subluxation”); several cases had associated lesions noted on subsequent imaging (e.g., Hill-Sachs and labral pathology) but these were not contemporaneously linked to ED radiographic adequacy in the records.

Overall, the majority of patients did not receive orthogonal post-reduction views, and inadequate imaging was seldom recognised or acted upon by resident doctors at the point of ED review.

## Discussion

This study demonstrates that most patients with anterior shoulder dislocation presenting to our emergency department did not receive adequate post-reduction radiographs. Only 23% of cases met the minimum standard of orthogonal views, and in over three-quarters of patients the adequacy standard was not achieved. Importantly, when post-reduction films were inadequate, this was seldom recognised or acted upon by resident doctors, with only two instances of recognition documented. This deficiency in fundamental imaging practices raises concerns about the potential for missed or delayed diagnoses of significant associated pathologies, such as glenoid fractures or Hill-Sachs lesions, which can significantly impact patient management and long-term outcomes [8,9]. 

The requirement for orthogonal post-reduction imaging is a basic principle of musculoskeletal radiography, ensuring that concentric reduction has been achieved and that associated fractures are not overlooked. Both the BESS guideline and our local hospital policy emphasise the need for anteroposterior and either axillary or scapular Y views following shoulder reduction [3]. In contrast to this clear guidance, our findings highlight a consistent practice gap, with most patients receiving only a single AP film. This discrepancy is notable given that national guidelines also address advanced imaging such as MRI/MRA for instability assessment, yet our data indicate that even the most fundamental radiographic standards are not being met [10,11].

The absence of explicit requests for two views in our series may have contributed to the high rate of inadequate imaging. While orthogonal radiographs are a basic principle in musculoskeletal practice and required by local policy, clearer specification on request forms could help ensure compliance. Responsibility lies with both requesting clinicians and radiographers, and incorporating mandatory wording into request protocols may reduce this gap.

The clinical implications of inadequate imaging are significant [12]. Failure to obtain orthogonal views risks missed malreduction or associated injuries, such as greater tuberosity fractures or Hill-Sachs lesions [8]. While our series did not document acute delays in discharge or repeat imaging within the ED, this reflects a failure to identify the inadequacy at the time, rather than an absence of risk. In practice, unrecognised inadequate imaging may result in delayed diagnosis, additional imaging at a later stage, inconvenience to patients, and inefficient use of resources [13]. The observation that residents frequently accepted inadequate films suggests uncertainty in image interpretation and potential over-reliance on clinical assessment of stability.

Several factors may contribute to this shortfall. Positioning patients in pain for axillary or scapular Y views is challenging, and time pressures in the ED can lead to shortcuts in imaging requests [14]. Radiographers may be reluctant to attempt technically difficult views without explicit instruction, and junior doctors may lack confidence in judging adequacy [5]. These barriers underline the importance of targeted education for both ED clinicians and radiographers, alongside reinforcement of departmental policy.

Our study has limitations. The sample size was small, limiting statistical precision, and data were drawn from a single centre. Adequacy was assessed using documentation and radiology records, which may be subject to reporting bias. We did not capture long-term outcomes to quantify whether missed malreductions or fractures directly affected patient management. In addition, imaging protocols and clinician familiarity may differ across centres, which could limit the wider applicability of our findings. Nevertheless, the consistency of our findings across consecutive cases suggests a genuine gap between guideline recommendations and current practice.

Future work should focus on implementing simple interventions such as educational sessions, checklists in imaging request software, or integration of orthogonal view reminders into electronic proformas. Re-audit following such measures would allow assessment of improvement. Given that inadequate imaging represents a fundamental patient safety concern, improving compliance with this basic standard should be prioritised before addressing more advanced imaging strategies.

## Conclusions

Most patients with anterior shoulder dislocation in our emergency department did not receive adequate post-reduction radiographs, with only a minority achieving the recommended orthogonal views. Inadequacies were rarely recognised by resident doctors, highlighting both a compliance gap with established guidelines and a training need in radiograph interpretation. Although repeat imaging was not obtained in the ED, the absence of recognition means the true impact on patient safety may be underestimated. Ensuring orthogonal post-reduction imaging is a basic, low-cost measure that reduces the risk of missed malreduction or associated injury. Targeted education for clinicians and radiographers, reinforcement of local policy, and re-audit after intervention are recommended to improve practice.

We believe the value of this study lies in showing that even fundamental principles may be overlooked in busy emergency settings, a gap between policy and practice that has wider relevance across healthcare.
